# Effects of posterior condylar osteophytes on gap balancing in computer-assisted total knee arthroplasty with posterior cruciate ligament sacrifice

**DOI:** 10.1007/s00590-017-2118-2

**Published:** 2018-01-04

**Authors:** Pornpavit Sriphirom, Chaiyaporn Siramanakul, Boonyawat Chanopas, Sirisuk Boonruksa

**Affiliations:** 1 Department of Orthopaedic Surgery, Rajavithi Hospital, Rangsit University, Bangkok, Thailand; 2 Department of Orthopaedic Surgery, Banphaeo Hospital (Sathorn Branch), Chan Road, Bangkok, 10120 Thailand; 3 Department of Orthopaedic Surgery, Medical Development Clinic, Bangkok, Thailand; 4 Department of Orthopaedic Surgery, Phraphuttabat Hospital, Saraburi, Thailand

**Keywords:** Computer-assisted surgery, Total knee arthroplasty, Posterior condylar osteophyte, Gap balancing

## Abstract

**Objective:**

Gap planning in navigated total knee arthroplasty (TKA) is a critical concern. Osteophytes are normally removed prior to gap planning, with the exception of posterior condylar osteophytes of the femur, which are removed after posterior condylar resection. This study investigated how posterior condylar osteophytes affect gap balancing during surgery.

**Methods:**

This prospective study was conducted on 40 primary varus osteoarthritic knees with a posterior condylar osteophyte that underwent TKA navigation. For all knees, computed tomography (CT) was performed to evaluate osteophyte position. The extension gap and flexion gap were determined under navigation using a tension device with a distraction force of 44 lb. The extension gap and flexion gap were measured before and after osteophyte removal.

**Results:**

This study revealed that the average osteophyte thickness after removal was 7.75 ± 5.34 mm. The average extension gap change was 0.64 ± 0.80 mm, and the average flexion gap change was 0.85 ± 1.12 mm. With respect to increases in the medial extension gap, lateral extension gap, medial flexion gap and lateral flexion gap, the average effects of posterior condylar osteophyte removal were 0.74 ± 0.81 mm, 0.53 ± 0.96 mm, 0.71 ± 0.97 mm and 1.00 ± 1.41 mm, respectively. Posterior condylar osteophyte thickness was also significantly associated with increases in the lateral extension gap (*R*^2^ = 0.107, *p* = 0.03), medial flexion gap (*R*^2^ = 0.101, *p* = 0.04) and lateral flexion gap (*R*^2^ = 0.107, *p* = 0.04).

**Conclusion:**

These results indicated that posterior condylar osteophytes of the femur affect gap balancing during TKA navigation.

## Introduction

The goal of total knee arthroplasty (TKA) is to intraoperatively equalize the extension and flexion gaps [1, 2]. The restoration of neutral coronal and sagittal limb alignment is also important [3–5]. Computer-assisted surgery TKA (CAS TKA) has provided excellent gap balancing and accurate positioning of the prosthesis [6, 7]. Therefore, the intraoperative gap balancing plan during TKA navigation is of critical concern for achieving all treatment goals [8]. Issa et al. [9] reported an intraoperative change of 17.5% in a polyethylene-bearing insert after patient-specific instrument (PSI) planning. This finding indicates that PSI-based templates cannot be used to calculate factors that contribute to gaps. Contributing factors that affect extension and flexion gaps include proximal tibial resection, downsizing of the femoral component, the posterior cruciate ligament and osteophytes [10–13]. Osteophytes are normally removed before the gap planning step during TKA navigation. In certain cases, soft tissue can be extremely tight, rendering it difficult to remove posterior osteophytes before gap measurement. A posterior condylar osteophyte of the femur is sometimes removed during flexion gap preparation or may not be removed at all, depending on the technique that is utilized. The purpose of this study was to investigate how posterior condylar osteophytes affect gap balancing during CAS TKA. Additionally, this study examined how osteophyte thickness affects gap changes.

## Methods

**Fig. 1:**
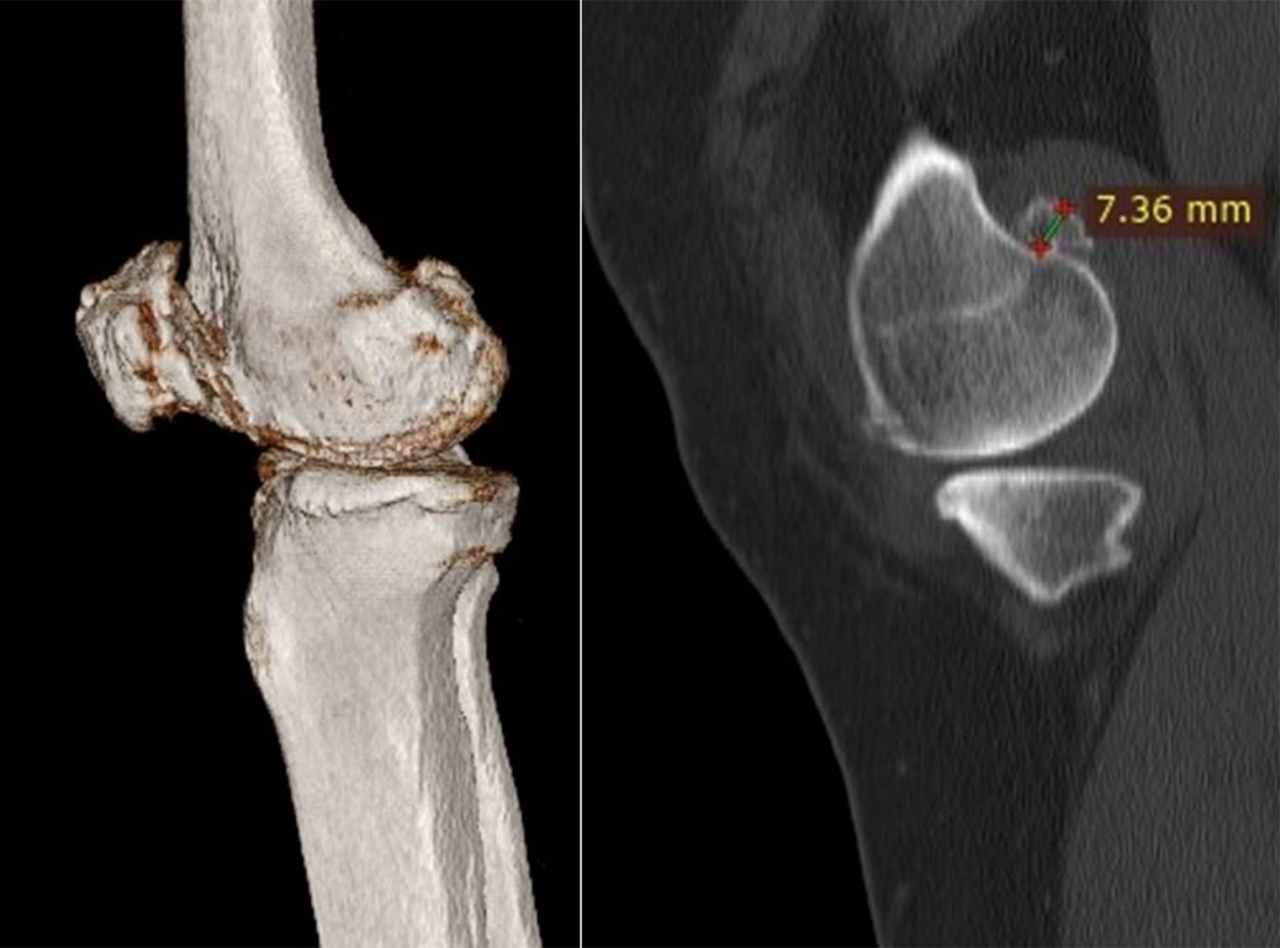
CT scans for preoperative evaluation of the posterior condylar osteophyte position

From June 2012 to June 2013, this prospective study was conducted on 40 varus osteoarthritic knees with a posterior condylar osteophyte of the femur that underwent primary TKA with a navigation system (Orthopilot 4.4, B. Braun Aesculap, Tuttlingen, Germany). The study received institutional review board approval from Banphaeo hospital. The participants gave informed consent for this study. In all cases, a mobile posterior stabilized (PS) prosthesis was used (e.motion-PS, B. Braun, Aesculap, Tuttlingen, Germany). The exclusion criteria were a varus deformity of more than 14°, a valgus knee deformity, a bony defect of more than 5 mm at the tibia or femur, and knee instability. For all knees, a preoperative hip–knee–ankle radiograph was obtained during full-leg standing for mechanical femorotibial angle measurement, and computed tomography (CT) scans were performed to evaluate osteophyte position (Fig. 1). The knees were exposed via a mini-midvastus approach using a tourniquet. The deep medial collateral ligament was released to expose the medial tibial plateau. The anterior and posterior cruciate ligaments were resected. Navigation registration was performed in stepwise fashion. The tibial cut was established first using a soft tissue management program. A proximal tibial osteotomy was performed under navigation, with each cut made perpendicular to the mechanical axis in the coronal plane and with 0° of posterior inclination along the sagittal plane. After proximal tibial resection, verification of the tibial cut was performed. The widths of the extension gap at 0° and flexion gap at 90° were measured, while the patella was reversed by utilizing a knee balancer (DePuy Orthopedics Inc., Warsaw, IN, USA) with a distraction force of 44 lb on both the medial and lateral sides under navigation [14]. The tension device was applied and maintained until constant measurements were obtained to reduce error that can result from creep elongation of surrounding soft tissues. During each measurement, the thigh was held, and the knee was aligned in the sagittal plane to eliminate the external load on the knee at 90° of knee flexion. The next step involved removing the posterior condylar osteophyte with a curved osteotome according to the positioning determined via CT. The patient was excluded from the study if a torn posterior capsule was found during osteophyte removal. A vernier caliper was used to measure the thickness of the posterior condylar osteophyte (in mm) from the inner cut surface to the outer surface that attached to the joint capsule. Extension and flexion gap widths were measured under navigation before and after removal of the posterior osteophyte of the femoral condyle to determine differences for comparative analyses.

### Statistical analysis

To determine the sample size, we performed a priori power analysis with significance levels for simple regression analysis set to an *α*-value of 0.05 and a power of 0.8. The appropriate sample size was 38 knees. Quantitative data were presented as the mean ± standard error of the mean, median and range. Paired *t* tests were performed to compare differences in the extension and flexion gaps before and after posterior condylar osteophyte removal. Simple regression analyses were performed to assess correlations between the thickness of the posterior condylar osteophyte and changes in the extension and flexion gaps. The threshold for statistical significance was *p* < 0.05, and SPSS for Windows (version 15.0, SPSS, Chicago, IL, USA) was used for statistical analyses.

## Results

The study revealed that based on vernier caliper measurements, the mean posterior condylar osteophyte thickness after removal was 7.75 ± 5.34 mm. All posterior condylar osteophytes were positioned on the medial side. The mean preoperative coronal alignment was 5.48° ± 3.53° varus. In all cases, the posterior capsule was intact after posterior condylar osteophyte removal. Gap changes after posterior condylar osteophyte removal are shown in Table 1. Both the extension and flexion gaps significantly increased (*p* < 0.05). The average extension gap change was 0.64 ± 0.80 mm, and the average flexion gap change was 0.85 ± 1.12 mm. The flexion gap increased by significantly more than the extension gap (*p* *=* 0.00) after posterior condylar osteophyte removal.

**Table 1:** Extension and flexion gap change after posterior condylar osteophyte removal

	Medial	*p* value*	Lateral	*p* value*
Extension gap change	0.74 ± 0.81	0.00	0.53 ± 0.96	0.00
Flexion gap change	0.71 ± 0.97	0.00	1.00 ± 1.41	0.00

* Paired *t* test

Table 2 shows the number of cases with gap increase and no gap increase after osteophyte removal. The overall incidence of gap increase was lower than no gap change except in medial extension gap.

**Table 2:** Number and percentage of cases with gap increase and no gap increase after posterior condylar osteophyte removal

Gap compartment	Number of cases with gap increase	Number of cases with no gap increase	Median of gap change (mm)	Range (mm)
Medial extension gap	21 (52%)	19 (48%)	1	1–3
Medial flexion gap	18 (45%)	22 (55%)	1	1–4
Lateral extension gap	15 (37.5%)	25 (62.5%)	1	1–5
Lateral flexion gap	18 (45%)	22 (55%)	1.5	1–5

**Fig. 2:**
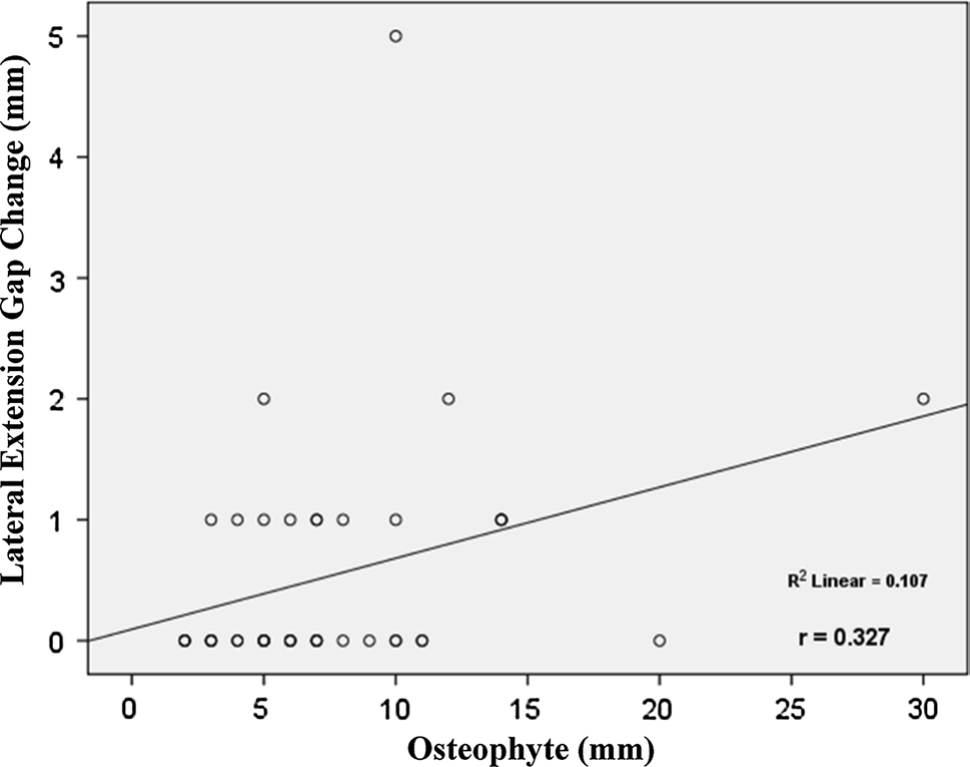
Correlation between the thickness of osteophyte and the lateral extension gap change. The thickness of osteophyte was positively correlated with lateral extension gap change

**Fig. 3:**
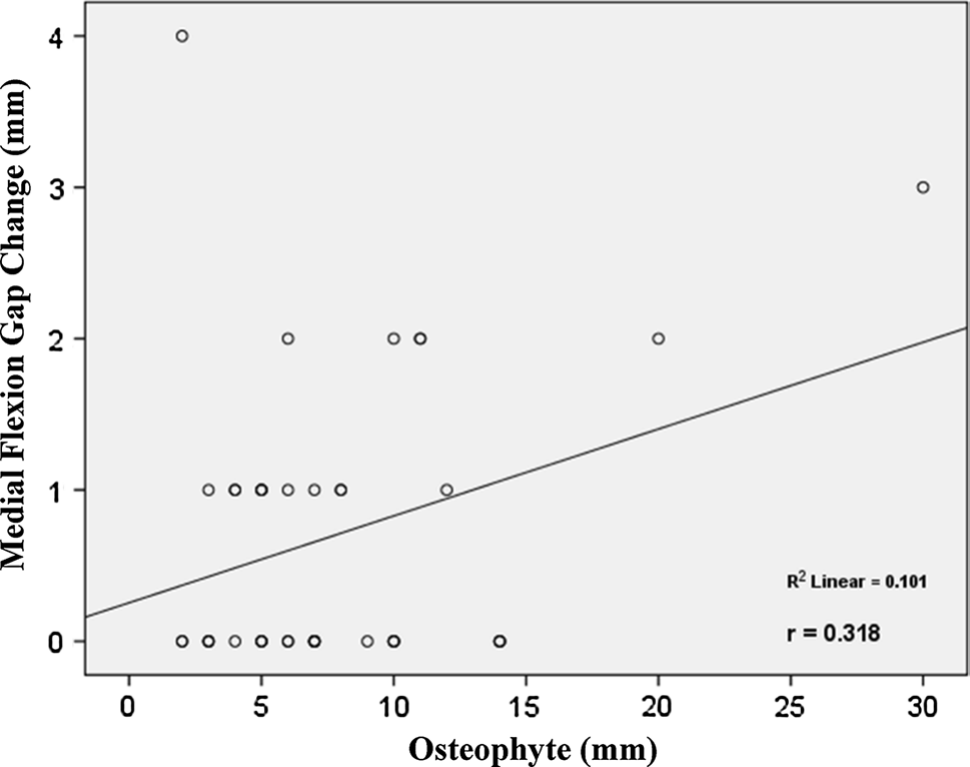
Correlation between the thickness of osteophyte and the medial flexion gap change. The thickness of osteophyte was positively correlated with medial flexion gap change

**Fig. 4:**
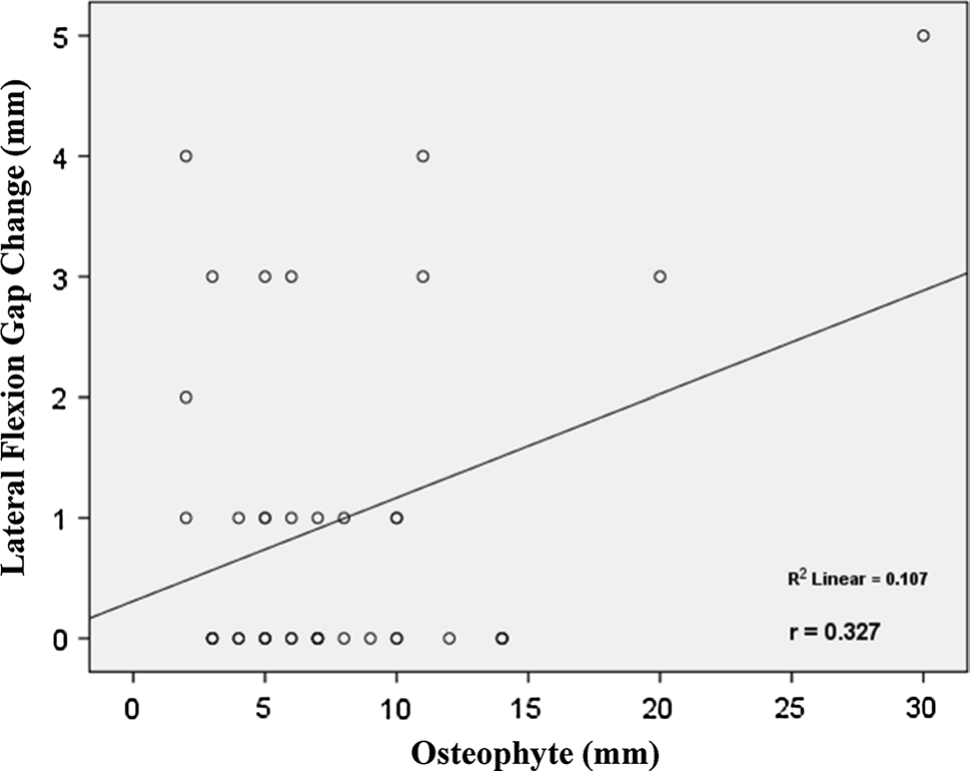
Correlation between the thickness of osteophyte and the lateral flexion gap change. The thickness of osteophyte was positively correlated with lateral flexion gap change

The study found significant relationships between the thickness of the posterior condylar osteophyte (mm) and increases in the lateral extension gap (*R*^2^ = 0.107, *p* *=* 0.03), medial flexion gap (*R*^2^ = 0.101, *p* *=* 0.04) and lateral flexion gap (*R*^2^ = 0.107, *p* *=* 0.04) (Figs. 2, 3, 4). The lateral extension gap change could be calculated using the expression 0.06 × osteophyte thickness (in mm) + 0.095. The medial flexion gap change could be calculated using the expression 0.06 × osteophyte thickness (in mm) + 2.55. The lateral flexion gap change could be calculated using the expression 0.09 × osteophyte thickness (in mm) + 0.31. However, no expression could be used to reliably predict the medial extension gap change.

## Discussion

In TKA, a posterior condylar osteophyte of the femur is relatively easy to remove after posterior condylar resection during the flexion gap preparation step. In particular, during CAS TKA with a gap balancing program, it is easier to remove posterior osteophytes after the 4-in-1 femoral cut rather than before posterior condyle resection, as the software requests. The modified gap technique involves starting with initial preparation of the extension gap, followed by matching of the flexion gap to the extension gap [15]. This technique minimizes the risk of joint line elevation; however, preparation of the flexion gap with resection of a posterior condylar osteophyte could potentially change the tension of the posterior capsule and therefore change the intended extension and flexion gap planning. Sugama et al. [16] found increases of 2.7 mm in the medial extension gap and 2.1 mm in the lateral extension gap after flexion gap preparation in PS TKA. However, they did not evaluate posterior condylar osteophyte-associated effects. In cruciate-retaining TKA, Minoda et al. [17] showed that the extension gap increased by approximately 0.4 mm after posterior condylar resection and by 0.6 mm after posterior condylar osteophyte removal. Recently, Seo et al. [18] evaluated the effects on the extension gap of posterior condylar resection with no or minimal osteophytes during PS TKA and found an increase of only approximately 1 mm in the extension gap. However, the aforementioned studies were performed using manual instruments, whereas computer navigation can be used to measure intraoperative gap changes with an accuracy of 1 mm [19]. The results of the current study revealed that both the extension and flexion gaps increased after posterior condylar osteophyte removal (by 0.64 and 0.85 mm, respectively). However, there was a greater mean increase in the flexion gap than in the extension gap, and the largest change occurred in the lateral flexion gap (1 mm). The actual navigation system rounds the measures to the nearest the whole number. That means that a 0.4-mm gap increase is rounded to 0 mm, while a 0.5-mm increase is rounded to 1 mm. Then, our findings show that the means of all gap changes will be rounded to 1 mm increase after osteophyte removal. Moreover, Table 2 shows the percentage of gap increase was lower than no gap change except in medial extension gap after osteophyte removal that means that the thickness of osteophyte should effect on the gap change. This study reveals relationships between osteophyte thickness and increases in the lateral extension gap, medial flexion gap and lateral flexion gap. It is believed that removal of a posterior condylar osteophyte should result in the relative lengthening of the posterior capsule and therefore an increase in both the extension and flexion gaps. However, there remains no clear explanation for why the largest change was observed for the lateral flexion gap when all posterior condylar osteophytes were on the medial side. Our findings are interesting but require additional investigation. Although there were a small number of gap changes after osteophyte removal, the magnitude of these changes was only approximately 1 mm. An additional 1 mm change in the extension gap after posterior condylar resection [18] could result in an increase of 2 mm in the extension gap. This phenomenon could lead to a change in a polyethylene insert from the intraoperative plan.

## Limitations

There were several limitations of this study. Few patients were included in this investigation. However, the sample size was sufficient based on an a priori power analysis. Valgus knees were not included in this study. Therefore, the study findings may not be applicable to valgus knees. Although posterior osteophyte volume would likely be a more relevant measure than osteophyte thickness, the sagittal dimension of posterior osteophytes is more easily measured via CT or plain film images in clinical practice. The effects of posterior condylar osteophyte removal on gap changes appeared to be relatively small; however, greater accuracy is required for gap planning in CAS TKA. It is hoped that the results of this study can be applied to provide benefits in the development of CAS TKA software and PSI templates to enable more precise gap planning.

## Conclusion

In conclusion, the removal of a posterior condylar osteophyte of the femur affects increases in both the extension and flexion gaps. This removal caused a greater increase in the flexion gap than in the extension gap, and posterior condylar osteophyte thickness was positively correlated with increases in all gaps except for the medial extension gap. The results of this study raise further concerns that CAS TKA or conventional TKA for an osteoarthritic knee with a large posterior condylar osteophyte of the femur can cause gaps to increase and become larger than specified in the gap balancing plan.
